# The Interplay Between Autophagy and RNA Homeostasis: Implications for Amyotrophic Lateral Sclerosis and Frontotemporal Dementia

**DOI:** 10.3389/fcell.2022.838402

**Published:** 2022-04-28

**Authors:** O. H. Houghton, S. Mizielinska, P. Gomez-Suaga

**Affiliations:** ^1^ Department of Basic and Clinical Neuroscience, Institute of Psychiatry, Psychology and Neuroscience, King’s College London, Maurice Wohl Clinical Neuroscience Institute, London, United Kingdom; ^2^ UK Dementia Research Institute at King’s College London, London, United Kingdom; ^3^ Departamento de Bioquímica y Biología Molecular y Genética, Facultad de Enfermería y Terapia Ocupacional, Universidad de Extremadura, Cáceres, Spain; ^4^ Centro de Investigación Biomédica en Red de Enfermedades Neurodegenerativas (CIBERNED), Madrid, Spain; ^5^ Instituto Universitario de Investigación Biosanitaria de Extremadura (INUBE), Cáceres, Spain

**Keywords:** autophagy, RNA, amyotrophic lateral sclerosis, frontotemporal dementia, RNA-binding proteins, C9orf72, stress granules, granulophagy

## Abstract

Amyotrophic lateral sclerosis and frontotemporal dementia are neurodegenerative disorders that lie on a disease spectrum, sharing genetic causes and pathology, and both without effective therapeutics. Two pathways that have been shown to play major roles in disease pathogenesis are autophagy and RNA homeostasis. Intriguingly, there is an increasing body of evidence suggesting a critical interplay between these pathways. Autophagy is a multi-stage process for bulk and selective clearance of malfunctional cellular components, with many layers of regulation. Although the majority of autophagy research focuses on protein degradation, it can also mediate RNA catabolism. ALS/FTD-associated proteins are involved in many stages of autophagy and autophagy-mediated RNA degradation, particularly converging on the clearance of persistent pathological stress granules. In this review, we will summarise the progress in understanding the autophagy-RNA homeostasis interplay and how that knowledge contributes to our understanding of the pathobiology of ALS/FTD.

## Introduction

Amyotrophic lateral sclerosis (ALS) is the most common form of motor neuron disease and is known to be clinically, genetically and pathologically linked to frontotemporal dementia (FTD), the second most common form of presenile dementia after Alzheimer’s disease. Thus, both disorders relate to one another on a disease spectrum for which there is currently no cure nor disease-modifying treatment available. A considerable number of studies have provided evidence for a link between the dysregulation of RNA homeostasis and defective autophagy in ALS/FTD. Furthermore, recent literature from the autophagy and RNA research fields has provided intriguing connections between these key cellular mechanisms, with implications for our understanding of ALS/FTD pathogenesis.

Autophagy is an evolutionarily conserved lysosomal catabolic pathway that ensures nutrient recycling and removal of unwanted substrates. Consequently, autophagy plays a crucial homeostatic role for maintaining healthy and functional cells, enabling adaptation to changing cellular demands and protection from stress. Even though most studies have focused on autophagy-mediated protein catabolism, accumulating data supports autophagy-mediated RNA degradation as an important additional cellular RNA quality control mechanism. RNA, RNA-binding proteins (RBPs) and RNA-protein complexes (such as RNA granules) can all be degraded by autophagy, although the mechanisms of these processes and their biological consequences have not yet been fully determined and this is now an active field of research. Furthermore, new non-conventional functions of autophagy machinery proteins impacting key aspects of RNA homeostasis have been revealed.

The dysregulation of RNA and protein homeostasis is a major contributor to ALS/FTD pathogenesis. Notably, abnormal accumulation of insoluble protein inclusions in neurons and neuroglia in affected brain regions is a key hallmark of ALS/FTD. These inclusions are frequently characterized by the presence of RBPs involved in RNA processing, such as TAR-DNA binding protein (TDP-43), and proteins from the autophagy machinery, like the selective autophagy receptor, p62. Moreover, ALS/FTD genetics have also uncovered a substantial number of genes involved in autophagy and RNA metabolism. Thus, autophagy and RNA homeostasis are two key dysregulated molecular processes and there is an emerging perspective of an interplay between them in disease.

In this review, we will first provide an overview of important aspects of ALS/FTD and autophagy and then present novel findings regarding conventional and non-conventional functions of the autophagy machinery in RNA catabolism. We will then summarize key evidence for defective autophagy in ALS/FTD, dissecting the relevance of disease-associated proteins in the different stages of this process and defective transcriptional and post-transcriptional regulation. Finally, we will discuss the emerging impact of the interplay between autophagy and RNA homeostasis with particular reference to the complexities in C9orf72-ALS/FTD.

## An Overview of Amyotrophic Lateral Sclerosis/Frontotemporal Dementia

ALS and FTD are characterized by progressive degeneration of neurons in affected areas of the nervous system: motor neurons in the motor cortex, brainstem, and spinal cord in ALS and cortical neurons in the frontal and temporal lobes in FTD (Abramzon et al., 2020). Degeneration in these areas results in the clinically observed symptoms of loss of motor control in ALS and behavioural and language dysfunctions in FTD. Despite being clinically distinct, symptoms of both diseases are regularly observed in the same person or segregating within affected families (Abramzon et al., 2020). FTD and ALS are also linked by a common pathological signature, with the vast majority of familial and sporadic cases of ALS and a significant proportion of FTD displaying pathological accumulation of the RBP TDP-43 into cytoplasmic inclusions in neurons and also in glial cells (Neumann et al., 2007). Apart from the clinical and pathological overlap, genetics also unifies both disorders. Classic familial inheritance is observed in 5%–10% of ALS cases and in 30%–50% of FTD cases; whereas some genes like *SOD1* or *MAPT* are predominantly linked to ALS or FTD, respectively, disease-causing variants in a number of genes have been identified in ALS/FTD families (see Table 1). Of particular note, in 2011, the discovery of a pathogenic G_4_C_2_ hexanucleotide repeat expansion in a non-coding region of *C9orf72* gene causing both ALS and FTD, strengthened the genetic link between these neurodegenerative disorders (DeJesus-Hernandez et al., 2011; Renton et al., 2011). The *C9orf72* mutation is the most common cause of familial ALS/FTD with around 40% and 25% of familial ALS or FTD carrying the *C9orf72* repeat expansion, respectively, and accounting for around 6% of sporadic cases in both as well (Majounie et al., 2012).

**TABLE 1:** Autophagy-related ALS/FTD genes and their putative direct or indirect functions in dysfunctional autophagy-RNA homeostasis interplay.

Gene Abbreviation	Gene name	Clinical presentation	Autophagy-related function & roles in autophagy-dependent RNA homeostasis	References
*SQSTM1*	Sequestosome 1 (p62)	ALS, FTD	Receptor for selective autophagy	***Selective autophagy*:** (Pankiv et al., 2007; Deng et al., 2020)
			Role in granulophagy	***Autophagy-dependent RNA catabolism*:** (Buchan et al., 2013; Guo et al., 2014; Ganassi et al., 2016; Chitiprolu et al., 2018; Turakhiya et al., 2018)
*OPTN*	Optineurin	ALS, FTD	Receptor for selective autophagy	***Selective autophagy*:** (Wild et al., 2011; Richter et al., 2016)
			Role in SG dynamics	***Autophagy-dependent RNA catabolism*:** (Kakihana et al., 2021)
*TBK1*	TANK-binding kinase 1	ALS, FTD	Modulation of the selective autophagy receptors optineurin and p62	***Autophagy (various)*:** (Sellier et al., 2016)
				***Selective autophagy*:** (Wild et al., 2011; Pilli et al., 2012; Matsumoto et al., 2015; Richter et al., 2016)
			Modulation of other autophagy proteins – AMPK (initiation), syntaxin-17 (autophagosome-lysosome fusion), SMCR8 (various roles)	***Autophagy initiation*:** (Zhao P. et al., 2018)
				***Autophagosome-lysosome fusion*:** (Kumar et al., 2019)
*C9orf72*	Chromosome 9 open reading frame 72	ALS, FTD	C9orf72 protein: Roles in autophagy initiation, maturation, lysosomal function, vesicular trafficking, and cytoskeleton organization	***Autophagy initiation*:** (Amick et al., 2016; Sellier et al., 2016; Sullivan et al., 2016; Ugolino et al., 2016; Webster et al., 2016; Yang et al., 2016; Liu et al., 2018; Zhang et al., 2018; Cali et al., 2019; Ho et al., 2019; Ji et al., 2020; Tang et al., 2020; Wang M. et al., 2020)
			Role in granulophagy	***Maturation, docking and fusion*:** (Farg et al., 2014; Sellier et al., 2016; Sivadasan et al., 2016; Webster et al., 2016; Yang et al., 2016; Aoki et al., 2017; Corbier and Sellier, 2017; Iyer et al., 2018; Shi et al., 2018; Su et al., 2020; Tang et al., 2020; Fumagalli et al., 2021; Nörpel et al., 2021)
			Form foci capable of sequestering RBPs and RNA	***Lysosomal Degradation*:** (Burd and Cullen, 2014; Amick et al., 2016; O’Rourke et al., 2016; Yang et al., 2016; Aoki et al., 2017; Amick et al., 2018; Corrionero and Horvitz, 2018; Shi et al., 2018; Zhang et al., 2018; Laflamme et al., 2019; Amick et al., 2020; Shao et al., 2020; Lall et al., 2021)
			*C9orf72* mutation derived DPRs:	***Transcriptional and post-transcriptional regulation*:** (Ugolino et al., 2016; Yang et al., 2016; Liu et al., 2018; Cunningham et al., 2020; Ji et al., 2020; McEachin et al., 2020; Shao et al., 2020; Wang M. et al., 2020)
			Alter SG dynamics and sequester RBPs	***Autophagy-dependent RNA catabolism*:** (Lee K.-H. et al., 2016; Boeynaems et al., 2017; Chitiprolu et al., 2018; Chew et al., 2019; Božič et al., 2021)
*SMN*	Survival of motor neuron	ALS	RBP	***Autophagy (general)*:** (Garcera et al., 2013; Custer and Androphy, 2014)
			Role in autophagy (specifics undetermined)	***Autophagy-dependent RNA catabolism*:** (Chitiprolu et al., 2018)
			Role in granulophagy	
*VCP*	Vasolin-containing protein	ALS, FTD	Role in regulation of autophagy initiation via Beclin-1	***Autophagy initiation*:** (Hill et al., 2021)
			Role in SG dynamics and granulophagy	***Autophagy-dependent RNA catabolism*:** (Buchan et al., 2013; Seguin et al., 2014; Wang et al., 2019; Gwon et al., 2021)
*VAPB*	Vesicle-associated membrane protein B	ALS	Modulates autophagy initiation and nucleation	***Autophagy (general)*:** (Larroquette et al., 2015)
				***Initiation*:** (Gomez-Suaga et al., 2017; Zhao Y. G. et al., 2018; Rimessi et al., 2020)
			Modulates selective ER-phagy via CALCOCO1	***Selective autophagy*:** (Nthiga et al., 2020)
			Mutant VAPB aggregates colocalise with TDP-43 and the SG protein TIAR1	***Autophagy-dependent RNA catabolism*:** (Tripathi et al., 2021)
*CHMP2B*	Charged multivesicular body protein 2B	FTD	ESCRT protein involved in sorting of cargoes and vesicular completion	***Autophagy maturation and transport*:** (Clayton et al., 2018; Ugbode and West, 2021)
*ALS2*	Alsin 2	ALS	Role in the endocytic pathway	***Autophagy maturation*:** (Otomo et al., 2003; Hadano et al., 2010)
*FIG4*	Factor-induced gene 4	ALS, FTD	Vesicle maturation and fusion	***Autophagosome maturation/degradation*** (Chow et al., 2009; Ferguson et al., 2009; Katona et al., 2011; Bharadwaj et al., 2016)
*SIGMAR1*	Sigma-1 receptor	ALS, FTD	Role in autophagosome-lysosome fusion	***Autophagosome-lysosome fusion/degradation*:** (Gregianin et al., 2016; Christ et al., 2019; Yang et al., 2019)
*CCNF*	Cyclin F	ALS, FTD	Role in autophagosome-lysosome fusion	***Autophagosome-lysosome fusion/degradation*:** (Lee et al., 2018)
*GRN*	Progranulin	FTD	Regulation of lysosomal biology and transcription of lysosomal genes	***Lysosomal degradation*** (Liu et al., 2015; Lui et al., 2016; Chang et al., 2017; Kao et al., 2017; Tanaka et al., 2017; Elia et al., 2019)
				***Transcriptional regulation*** (Tanaka et al., 2017)
*UBQLN2*	Ubiquilin 2	ALS, FTD	Role in lysosomal degradation	***Lysosomal degradation*** (Şentürk et al., 2019; Wu et al., 2020)
			Localises to SGs and regulates FUS recruitment	***Autophagy-dependent RNA catabolism*** (Alexander et al., 2018; Dao et al., 2018)
			Mutants causes changes to FUS-SG interactions	
*SPG11*	Spatascin	ALS	Cytoskeletal component contributing to lysosomal reformation	***Lysosomal reformation*** (Chang et al., 2014)
*TARDBP*	TAR DNA binding protein	ALS, FTD	RBP and SG protein	***Transcriptional and post-transcriptional control*** (Bose et al., 2011; Ling et al., 2013; Zhang T. et al., 2014; Xia et al., 2016)
			Post-transcriptional regulation of key autophagy-related genes	***Autophagy-dependent RNA catabolism*** (Dewey et al., 2011; Gopal et al., 2017; Ding et al., 2021)
			Transcriptional regulation of autophagy-related genes through the transcription factor FOXO	
			Mutants causes changes to SG dynamics	
*FUS*	FUS	ALS, FTD	RBP and SG protein	***Post-transcriptional control*** (Arenas et al., 2021)
			Post-transcriptional regulation of key autophagy-related genes.	
			Modulation of the formation and maturation of autophagosomes.	***Autophagy initiation*** (Soo et al., 2015; Ling et al., 2019)
			Mutants causes changes to SG dynamics	***Autophagy-dependent RNA catabolism*** (Baron et al., 2013; Ryu et al., 2014; Murakami et al., 2015; Patel et al., 2015)
*hnRNPA1*	Heterogeneous nuclear ribonucleoprotein A1	ALS, FTD	RBP and SG protein	***Post-transcriptional control*** (Ji et al., 2019)
			Post-transcriptional regulation of Beclin1	
*ANXA11*	Annexin A11	ALS	RBP; tether between RNA granules and lysosomes for transportation	***Transport/autophagy-dependent RNA homeostasis*** (Liao et al., 2019; Nahm et al., 2020)
			Mutants cause SG disassembly	

The findings that sporadic and familial ALS/FTD are phenotypically and pathologically indistinguishable and that pathogenic variants in many ALS/FTD-linked genes are described in patients without a family history of disease highlight the value of studying the monogenic, heritable forms (Shepheard et al., 2021). Although ALS/FTD-associated genes do not fit easily into a single cellular pathway, they direct the focus to two key cellular processes: RNA metabolism and autophagy degradation, as many of the ALS/FTD-linked genes determined are implicated in these biological pathways. The first category of ALS/FTD-linked genes fall into those encoding for RBPs, like *TARDBP* (Sreedharan et al., 2008), *FUS* (Vance et al., 2009), *hnRNPA1* (Kim et al., 2013), *ANXA11* (Smith et al., 2017) and *SMN* (Blauw et al., 2012), implicated in RNA metabolism (see Table 1). RBPs bind to RNA at specific sequences or secondary structures to facilitate several steps of the RNA life cycle, both in the nucleus and cytoplasm. Under physiological conditions, the majority of ALS/FTD-associated RBPs are largely nuclear; however, under pathological conditions, such as when they are affected by ALS/FTD-associated mutations, they are often mislocalised to the cytoplasm and form large insoluble inclusions (Maziuk et al., 2017; Nussbacher et al., 2019).

Mislocalisation and aggregation of mutant RBPs impact their normal functions regulating RNA metabolism. This can result in a loss of function effect on their respective nuclear or cytoplasmic mRNA targets, but aggregation of ALS/FTD-related RBPs can also favour pathological interactions and sequestration of other RBPs or RNA (Nussbacher et al., 2019). An additional gain of function patho-mechanism may derive from the formation of aberrant RNA granules. This mechanism relies on the intrinsic aggregation-prone properties of RBPs; apart from RNA recognition motifs and nuclear import and export sequences, a feature of all RBPs is the presence of low complexity domains (LCDs). These domains allow transient multivalent interactions facilitating the formation of membraneless RNA-protein complexes, such as stress granules (SGs), which require the capacity to form and dissolve rapidly. Many ALS/FTD-causing mutations in RBPs occur in LCDs, increasing the tendency to form poorly dynamic and solid-like complexes which may form the seed for subsequent aggregation (reviewed in Mann and Donnelly, 2021; Solomon et al., 2021). Additionally, mutations in the stress granule components TIA1 and ataxin-2 (ATXN2) have also been associated with ALS (Polymenidou et al., 2011; Sephton et al., 2011; Mackenzie et al., 2017). As already noted, the most frequent RBP which aggregates in ALS/FTD is TDP-43 although mutations in *TARDBP* itself only underly 1%–4% of ALS/FTD cases, showing that alternative mechanisms can drive RBPs to aggregate (Sreedharan et al., 2008). Similarly, inclusions of the RBP FUS are found in brain tissue of some patients with ALS/FTD both with and without FUS mutations (Vance et al., 2009; van Langenhove et al., 2010).

A second category of ALS/FTD-linked genes fall in those encoding for proteins involved in the autophagy-lysosomal pathway (see Table 1). The products of these genes, such as those belonging to the autophagy machinery, like ubiquilin-2 (gene: *UBQLN2*) (Deng et al., 2011), optineurin (*OPTN*) (Maruyama et al., 2010), and p62 (*SQSTM1*) (Fecto et al., 2011; Rubino et al., 2012), are also identified in both neuronal and glial inclusions in *post-mortem* ALS/FTD patient tissue from both familial and sporadic cases, and sometimes colocalise with the above mentioned RBPs (Ramesh and Pandey, 2017). While this is also the case for two genes causing FTD (*CHMP2B* (Skibinski et al., 2005) and *GRN*), mutations in the *MAPT* gene predominantly lead to FTD with a different brain pathology, characterized by abnormal hyperphosphorylated tau filaments. Tauopathy is a feature of many diseases and, while autophagy also seems to be a key player in dealing with tau aggregates (Spillantini and Goedert, 2013), we will not discuss this within this review.

Similar to other ALS/FTD genes, the brain pathology of *C9orf72* ALS/FTD patients shows typical TDP-43 and p62 inclusions in neurons and glial cells (Al-Sarraj et al., 2011; Murray et al., 2011; Cooper-Knock et al., 2012). However, *C9orf72* patients additionally show unique RNA and protein pathologies stemming from the nature of the mutation. The large intronic repeat expansion mutation in *C9orf72* is transcribed into sense and antisense repeat RNA which form aggregates termed RNA foci in neurons and glia (Mizielinska et al., 2013). Although in a non-coding genomic region, the sense and antisense repeat RNA can also be translated via an unconventional mechanism into dipeptide-repeat proteins (DPRs) which form protein aggregates in *C9orf72*-ALS/FTD patient brain and spinal cord (reviewed in Balendra and Isaacs, 2018). The mutation also causes haploinsufficiency of the encoded C9orf72 protein itself. Research is revealing multiples roles for the C9orf72 protein in autophagy, and although its loss does not seem to be the dominant mechanism for neurodegeneration, recent literature supports a model where a combination of *C9orf72* haploinsufficiency and gain of function repeat RNA and DPR toxic mechanisms drive disease pathogenesis via a synergistic effect (reviewed in Shi et al., 2018).

Thus, ALS and FTD genetics and pathology have a strong association with dysfunction of RNA homeostasis and autophagy pathways and indicate them as key disease mechanisms that warrant further review.

## Autophagy

Autophagy is particularly important in neurons and neuroglia where unique autophagy signalling and functions operate (Stavoe and Holzbaur, 2019; Belgrad et al., 2020; Sung and Jimenez-Sanchez, 2020). **Neuronal autophagy** is temporally and spatially fine-tuned to face their post-mitotic and long-lived nature and to accommodate their highly polarized structure. In neurons, autophagy plays a key role maintaining neuronal homeostasis as the major degradative pathway for the clearance of larger targets, such as aggregates and dysfunctional organelles. Furthermore, autophagy regulates specific neuronal functions, such as neurodevelopment, including axonal outgrowth and synapse formation, and neuronal activity and plasticity, which underlies the unique function of neurons to pass signals along neuronal networks and enables nervous system functionality. Unsurprisingly, defective neuronal autophagy has been widely linked to proteotoxic stress, aging and neurodegeneration (Stavoe and Holzbaur, 2019).

Neuroglia, comprising microglia, astrocytes, and oligodendrocyte lineage cells, are the other major brain cell types and in recent years have emerged as key players in brain development, physiology and metabolism. However, functions of **autophagy in glial cells** are less well known than in neurons (Strohm and Behrends, 2020). For example, in microglia, the resident mono-phagocytic cells in the CNS, autophagy participates in immune-related processes such as inflammasome activation (Houtman et al., 2019) and has been shown to be crucial for synaptic homeostasis in mice (Kim et al., 2017). Astrocytes, which help neuronal survival via the release of neurotrophic factors (Li et al., 2019), secrete bioactive molecules in extracellular vesicles in a process that involves divergent secretory organelles, including lysosomes and secretory autophagic vesicles (Verkhratsky et al., 2016), as further explained later. Lastly, in oligodendrocytes, autophagy is involved in myelination, which is one of the crucial functions of these cells (Belgrad et al., 2020). As briefly exemplified here, autophagy contributes to the biology of different cell types in the brain, and thus understanding how autophagy differs or is differentially regulated in neurons and neuroglia is an area of research that warrants the increasing attention it is receiving (Fleming et al., 2022).

However, across all cell types, the autophagy process shares general common features, reviewed here. Three distinct autophagy pathways can be described depending on a cargo’s delivery route to the lysosome: chaperone-mediated autophagy (CMA), microautophagy, and macroautophagy. CMA utilizes cytosolic chaperone proteins, typically the heat shock protein family A (Hsp70) member 8 (HSPA8/HSC70), to bind soluble proteins and direct them to the lysosome. CMA protein targets contain a KFERQ-like recognition motif and are delivered across the lysosomal membrane one-by-one by the lysosomal-associated membrane protein 2A (LAMP2A), which acts as a receptor to translocate them to the lysosomal lumen for degradation (Tekirdag and Cuervo, 2018). During microautophagy cytoplasmic entities destined for degradation are directly taken up by lysosomes or late endosomes (endosomal microautophagy). Although it is the least studied form of autophagy, microautophagy has now proven able to be selective (reviewed in Tekirdag and Cuervo, 2018).

**FIGURE 1:**
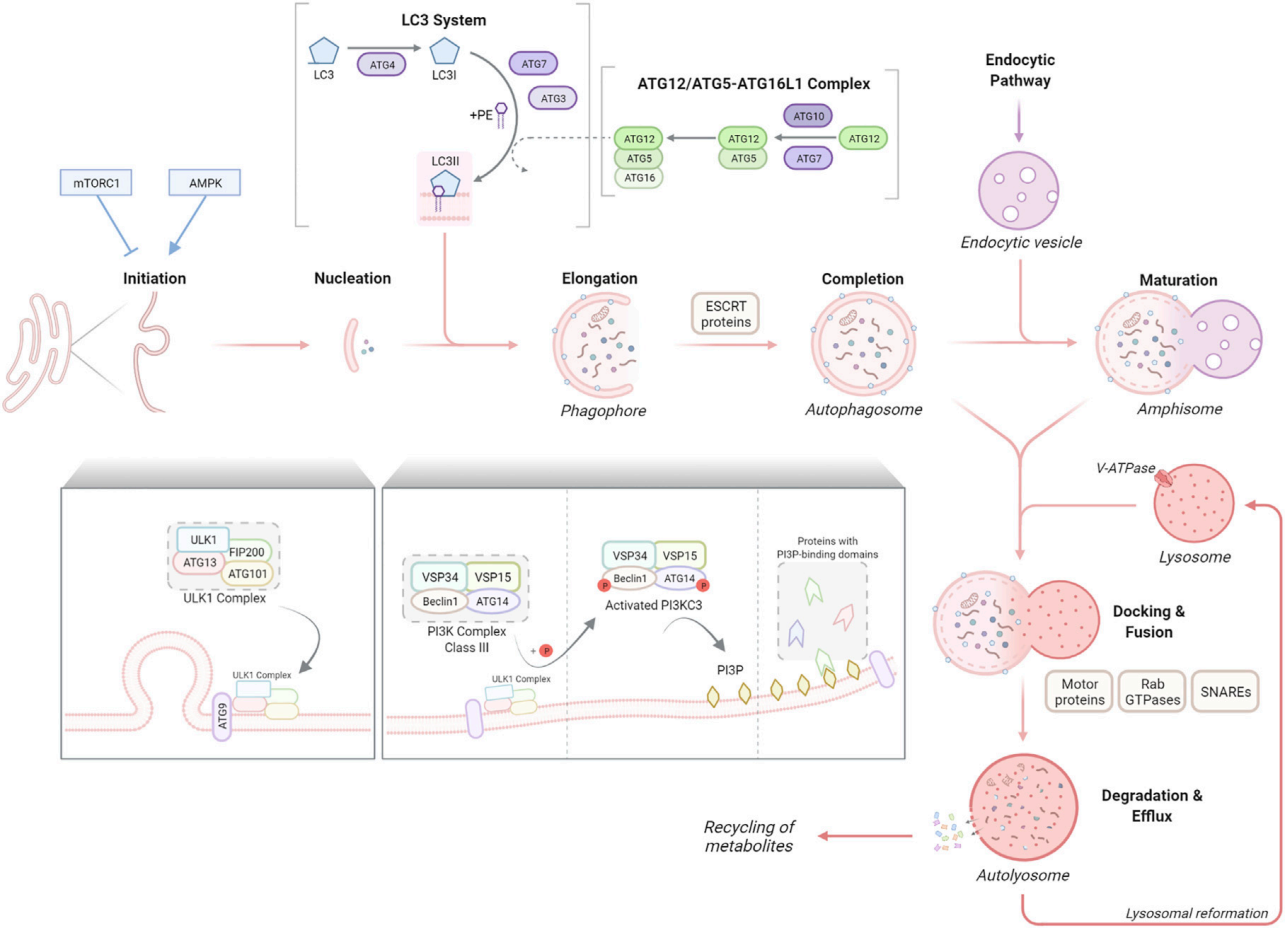
The stages of macroautophagy (autophagy). Autophagy initiation is tightly regulated commonly by two well-known kinases, mTORC1 and AMPK1, which inhibit or promote the activity of the ULK1 complex respectively. The ULK1 complex, consisting of ULK1, ATG13, FIP200 and ATG101, translocates to the autophagosome formation site with the transmembrane spanning ATG9 to stimulate nucleation of the phagophore membrane. The ULK1 complex activates the PI3K complex class III, formed of VSP34, VSP15, Beclin1 and ATG14, resulting in the production of PI3P and recruitment of proteins with PI3P-binding domains. The phagophore elongates, sequestering autophagic cargo for degradation. This elongation process is regulated by two ubiquitin-like reactions, the ATG12/ATG5–ATG16L1 complex formation and the conjugation of LC3 to the lipid anchor PE to form LC3-II, facilitated by ATG7 and other proteins. ESCRT proteins enable the elongation and fusing of phagophore edges to form the autophagosome which can undergo further maturation through fusion with endocytic vesicles to form amphisomes. Motor proteins, RAB GTPases and SNAREs facilitate the fusion of autophagosomes/amphisomes with lysosomes to form autolysosomes, within which cargo is degraded into metabolites and released for cellular recycling.

Macroautophagy, herein referred to as autophagy, is the most prevalent and well-characterized. Since it is by far the most studied autophagic pathway in the context of RNA catabolism and ALS/FTD, we will focus on this process. Autophagy involves *de novo* formation of cytoplasmic double-membrane organelles termed autophagosomes. Autophagosomes sequester cellular components, undergo further maturation steps and finally fuse with lysosomes leading to the degradation of the autophagic cargo by hydrolases. The resultant membranes and nutrients are recycled back to the cytosol for reuse (Figure 1). Thus, each stage of the autophagy process, with its own set of regulatory factors, provide a potential point of dysfunction in disease. Here we provide more details on the autophagy steps implicated in ALS/FTD pathogenesis, providing a context to the disease-associated changes reviewed later.

The signalling mechanisms leading to autophagy activation under different types of cellular stressors have been extensively investigated (Yu L. et al., 2018). Two well-known kinases are implicated in the control of this tightly regulated the **initiation** process: the nutrient sensor, mTOR (mammalian target of rapamycin) complex 1 (mTORC1) and the energy-sensing AMP-activated protein kinase (AMPK), whose inhibition or activation, respectively, triggers autophagy. The ULK1 (Unc-51-like autophagy-activating kinase) complex, which comprises ULK1 itself and ATG13, FIP200 (FAK family kinase interacting protein of 200 kDa; also known as RB1CC1) and ATG101, integrates upstream mTORC1 and AMPK signalling to coordinate the induction of the first steps of the autophagy process (Saxton and Sabatini, 2017).

To initiate autophagosome **nucleation**, the active ULK1 complex, together with the only multiple transmembrane-spanning ATG protein, ATG9, are translocated to autophagosome formation sites (Mercer et al., 2018); these have been described at different cellular compartments including the plasma membrane, Golgi, mitochondria, lipid droplets, but in particular at the ER (reviewed by Gómez-Sánchez et al., 2021). This translocation is followed by the recruitment of the VPS34/class III PI3K complex (comprising VPS34/PIK3C3, VPS15, Beclin1, and ATG14). Through phosphorylation of members of this complex, the ULK1 promotes PI3KC3 activity, generating the phospholipid PI3P (phosphatidylinositol 3-phosphate) to enable vesicle **elongation**. PI3P incorporation promotes the recruitment of proteins with PI3P-binding domains, such as FYVE domain-containing proteins (e.g. ALFY, autophagy FYVE-linked protein), which helps in the maturation of the new structure, the phagophore.

Two ubiquitin-like protein cascades, which are highly conserved, are required for the next steps. Two ubiquitin-like proteins, ATG12 and LC3 are conjugated to ATG5 or the lipid phosphatidylethanolamine (PE), respectively. Similar to ubiquitin, covalent conjugation of these proteins is achieved through a cascade of activities that are catalysed by E1 activating enzymes, E2 conjugating enzymes, and E3 ligases (Mizushima, 2020). Briefly, the ATG4 protease cleaves LC3 by removing C-terminal amino acids and exposing a glycine residue. This glycine is used in the first cascade mediated by the E1-like ATG7, followed by ATG3, an E2-like enzyme. Next, in the second cascade, the ATG12/ATG5–ATG16L1 complex, (elongation complex), is formed, involving the conjugation of ATG12 to ATG5 by the E1-like ATG7 and the E2-like ATG10 and the association of ATG16L1 with ATG5. The elongation complex, considered to have E3-like activity, determines the lipidation site of LC3 a process required for the association of LC3 with the autophagosomal membrane (Fujita et al., 2008). During this, LC3 is covalently bound to PE in the membrane (Mizushima, 2020). As the phagophore expands, LC3-PE (also known as LC3-II) is incorporated into the membrane. Consequently, the level of LC3-II can be used to determine the number of autophagosomes in cells (Klionsky et al., 2021). Finally, the edges of the phagophore fuse (vesicle **completion**) to form the autophagosome, which engulf the cargo for degradation. The endosomal sorting complexes required for transport (ESCRT) machinery is required for this step (Takahashi et al., 2018; Flower et al., 2020). Membrane-bound LC3-PE controls critical steps during the autophagy process apart from the autophagosome closure, such as movement of the autophagosomes or its fusion with the lysosomes, and degradation of the autolysosome inner membrane (Mercer et al., 2018). LC3 is also a key molecule for the binding to adapter proteins in **selective autophagy,** as further reviewed later.

Autophagosomes undergo fusion with endolysosomal compartments, including early endosomes, multivesicular bodies and late endosomes to form amphisomes (**maturation**). Autophagosomes/amphisomes are transported along microtubules to the perinuclear region where they fuse with lysosomes to form autolysosomes. The steps of **docking and fusion** involve a large set of molecules, including cytoskeleton components and related motor proteins, Rab GTPases, SNAREs and other proteins (Kriegenburg et al., 2018). Most of the molecular machinery that is responsible for these events involve proteins that are shared with the endocytic pathway (Mercer et al., 2018). Finally, the enclosed cargo is **degraded** by lysosomal hydrolases, allowing for the release and **recycling** of the newly generated metabolites and lysosomal reformation. An acidic environment in the lysosomal lumen, generated by the vacuolar H^+^ ATPase (V-ATPase) at the lysosomal membrane, is essential for this degradation (Yu L. et al., 2018).

Autophagy is also controlled at the **transcriptional regulation** level (di Malta et al., 2019). The transcription factor TFEB (transcription factor EB) and related MiTF/TFE (microphthalmia-associated transcription factor/transcription factor E) family members recognize and bind to coordinated lysosomal expression and regulation (CLEAR) motifs in the promoter region of many lysosomal and autophagy genes, leading to an increase in their transcription and a global enhancement of autophagy flux (Sardiello et al., 2009; Palmieri et al., 2011; Settembre et al., 2012; Martina et al., 2014; Ploper et al., 2015). We here highlight the role of the master regulator TFEB. TFEB shuttles between the nucleus and the cytoplasm, which is mainly regulated by mTORC1 and Rag GTPases on the lysosomal membrane (Puertollano et al., 2018). In response to amino acid signals, Rag GTPases recruit TFEB to the lysosome where it is phosphorylated by mTORC1, inhibiting its nuclear translocation and activation (Puertollano et al., 2018). Therefore basally, TFEB remains in its inactive phosphorylated form in the cytosol. Upon nutrient deprivation, or other cellular stressors, such as lysosomal damage or oxidative stress (Jia et al., 2018; Wang H. et al., 2020), TFEB gets dephosphorylated (activated) and translocates into the nucleus. Here, it enhances the expression of important genes for the autophagy-lysosomal pathway such as lysosomal proteins, for example, LAMP1/2 and hydrolases, and key proteins of the autophagy machinery such as beclin1 or p62 (Puertollano et al., 2018).

New studies have provided evidence for an additional regulatory layer of the autophagy process at the **post-transcriptional level**, governed by RBPs which regulate the processing and translation of specific autophagy-related transcripts (Sakellariou et al., 2021). Recently, noncoding RNAs have also been added to this regulatory function, furthering the interplay of autophagy and RNA (Xu et al., 2017; Yang et al., 2017; Zhao et al., 2020). However, although post-transcriptional/co-translational regulation of autophagy has received increasing attention in recent years, it is still poorly understood.

## Autophagy-Dependent RNA Catabolism

**FIGURE 2:**
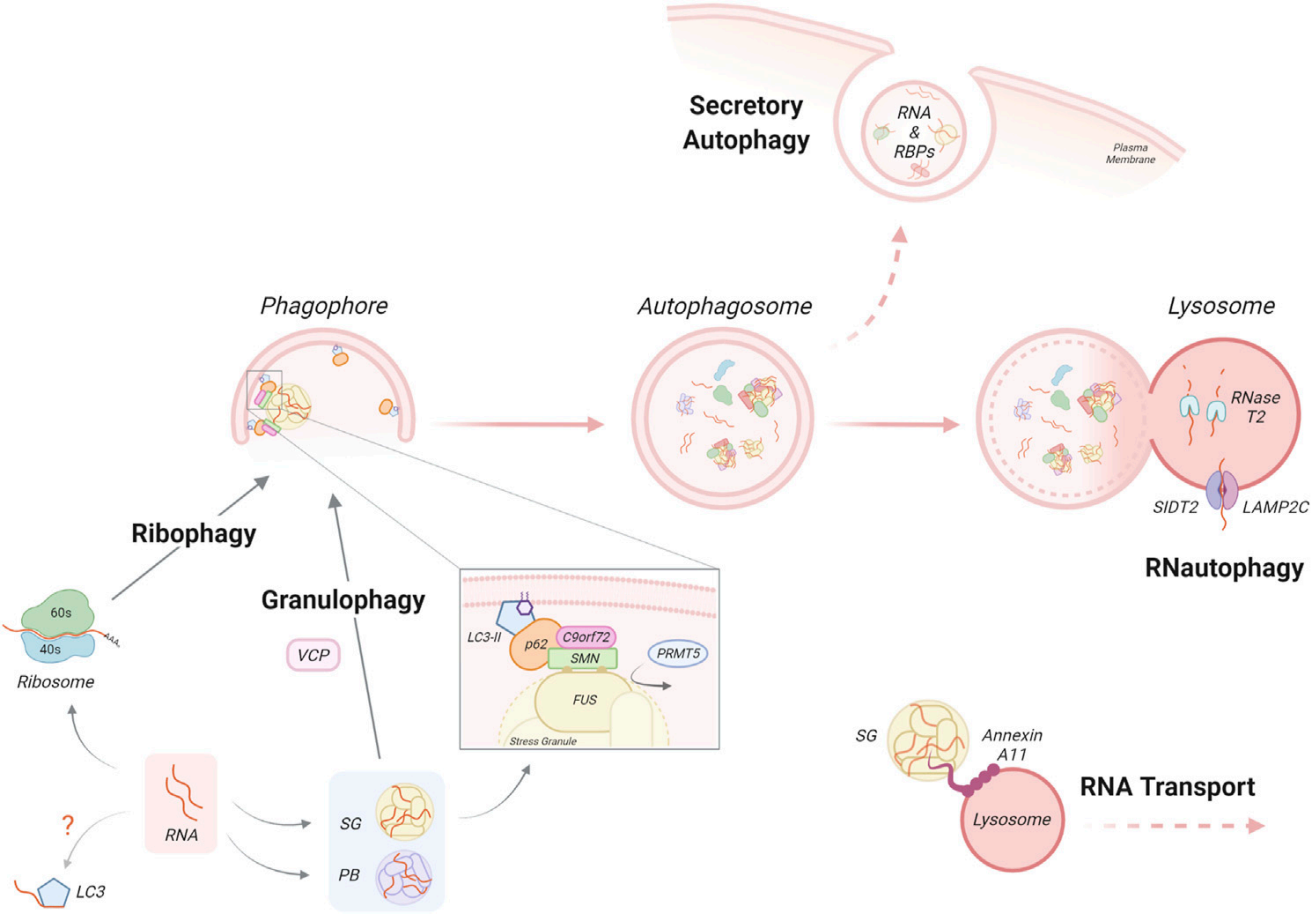
Autophagy-dependent RNA homeostasis. Autophagy-dependent RNA catabolism involves the degradation of RNA, RNA-binding proteins and RNA granules within the lysosomal lumen by RNases such as RNase T2 and lysosomal acidic hydrolases. Through **RNautophagy**, mRNA molecules are taken up directly into the lysosome through SIDT2 or LAMP2 proteins, in an ATP-dependent manner. Unbound RNA can also directly interact with LC3 via its arginine-rich motif however the impact of this requires further investigation. **Ribophagy** is a mechanism for autophagy dependent ribosome clearance by which ribosomes are engulfed within the autophagosome and delivered to the lysosome for degradation. mRNA can associate with RNA-binding proteins and accumulate in dynamic RNA granules such as stress granules (SG) or P-bodies (PB). **Granulophagy** is the process by which SGs are recruited for selective autophagy, which may involve the protein VCP. An additional mechanism of SG recruitment has been proposed, by which the PRMT5-dependent symmetric arginine methylation of SG components, such as FUS, allows for their recognition by the p62/C9orf72 complex via the SMN protein. Autophagy-dependent RNA catabolism is also impacted by the transport and secretion of RNA/RNA complexes. **RNA transport** involves SGs and other RNA complexes hitchhiking on motile vesicles, a process that involves the docking of RNA granules via molecular tethers. Notably, the ALS-associated RBP Annexin A11 is a component of SGs and can also bind lysosomes that are transported along microtubules. The autophagy machinery is also involved in the non-canonical autophagy function of **secretory autophagy**. By this process, the contents of autophagosomes, which may contain RNA and RBPs, bypass degradation and instead are directly secreted from the cell.

Apart from the conventional RNA decay pathways (reviewed by Houseley and Tollervey, 2009; Tatosyan et al., 2020), autophagy-mediated RNA degradation complements these quality control mechanisms to remove obsolete/defective RNA molecules. Additionally, RBPs and RNA-protein complexes, such as RNA granules, are also autophagy substrates, indirectly affecting RNA homeostasis. RNA degradation by autophagy was initially suggested in early observations from the 80–90s (Sameshima et al., 1981).These pioneering studies demonstrated increased RNA degradation during starvation-induced autophagy. In agreement with a lysosomal RNA degradation pathway, several acid ribonucleases (RNases) have been identified within lysosomes (Schröder et al., 2010). Interestingly, loss-of-function mutations in the RNase *RNASET2* leads to familial cystic leukoencephalopathy and displays lysosomal accumulation of ribosomal RNA in neurons and undigested substrates in microglia (Haud et al., 2011; Hamilton et al., 2020). Although the role of autophagy-dependent RNA catabolism has not been fully characterized, recent studies have described three different mechanisms - RNautophagy, ribophagy and granulophagy (Figure 2).

Uptake of mRNA molecules into lysosomes by the autophagy mechanism of **RNautophagy** does not require the formation of a double-membrane vesicle but instead resembles CMA (Schröder et al., 2010; Haud et al., 2011; Hamilton et al., 2020). During RNautophagy, the uptake occurs by direct binding of RNA to the lysosomal membrane proteins LAMP2C or SIDT2 (SID1 transmembrane family member 2), which act as nucleic acid receptors in an ATP-dependent manner (Fujiwara et al., 2013). The cytoplasmic region of LAMP2C/SIDT2 binds to RNA/DNA via its arginine-rich motif and possesses binding preference for stretches of poly-guanosines (Fujiwara et al., 2015; Hase et al., 2015; Hase et al., 2020). This binding may be regulated by other RNA-interacting proteins such as RBPs or DNA-binding proteins in the cytosol. Indeed, the ALS/FTD linked RBP hnRNPA1 was found to interact with LAMP2C in an RNA-dependent manner, in addition to a range of other RBPs (Fujiwara et al., 2013). *In vitro* studies have identified other lysosomal membrane proteins as RNA interactors, such as the mammalian CMA effector HSPA8 and the yeast lysosomal membrane V-ATPase subunit Vma1; however, the biological significance of these findings in terms of RNA-mediated autophagy degradation has not yet been determined (Castello et al., 2012). Indeed, further work is required to understand the substrates, sensors and regulation in RNautophagy. At present, the relative contributions of conventional RNA decay and this autophagy-mediated pathway in mRNA degradation are unclear.

Ribosomal RNA (rRNA) is the primary component of ribosomes and represents about 80% of total cellular RNA (Darnell, 1968; Warner, 1999; Weinberg et al., 2016). There are several pathways of eukaryotic ribosomal RNA decay (reviewed in Inada, 2020). However, ribosomes can also be targeted and removed by autophagy (An and Harper, 2020). Indeed, in electron microscopy studies, ribosomes are frequently detected inside autophagic structures in starved cells, leading to the general notion that ribosomes are non-selective cargo during bulk autophagy (Eskelinen et al., 2011). However, a growing body of evidence suggests that selective autophagic mechanisms directed to ribosomes (**ribophagy**) are employed in both yeast and mammalian cells (Beese et al., 2020). In mammalian cells, the protein NUFIP1 (nuclear fragile X mental retardation-interacting protein 1) has been identified as a ribophagy receptor for the 60S subunit, interacting with LC3B and ribosomes in response to nutrient stress (Wyant et al., 2018). However, a recent work has challenged this role, finding that NUFIP1 deletion has no effects on ribophagy flux (An et al., 2020). Indeed, a study from the same group has suggested that the overall contribution of ribophagy to ribosome homeostasis during nutrient stress is small relative to other pathways, such as the ubiquitin-proteasome system (An and Harper, 2018; An et al., 2020).

mRNAs exiting translation often accumulate in distinct cytoplasmic RNA-protein complexes, such as SGs and P-bodies. These are cytoplasmic membraneless organelles comprised of RNA and RBPs with key roles in the regulation of gene expression and cellular homeostasis (Alberti and Carra, 2018). SGs are assembled during a stress response, when global translation initiation is inhibited; thus, they are transient, reversible structures and their formation constitutes an adaptation mechanism for maintaining RNA metabolism homeostasis during unfavourable environmental conditions. Indeed, several studies have shown a pro-survival role for SGs (Reineke and Neilson, 2019; Samir et al., 2019). Interestingly, recent evidence suggests that translation of mRNAs can take place at the boundary of SGs, arguing against an exclusive role for SGs in inhibition of protein synthesis (Mateju et al., 2020). Physiologically, once the stress is resolved, SGs can be cleared by several mechanisms including disassembly by molecular chaperones (Ganassi et al., 2016; Mateju et al., 2017; Mediani et al., 2021), clearance by the ubiquitin–proteasome system (Turakhiya et al., 2018) and by selective autophagy – thus termed **granulophagy** (recently reviewed in Alberti and Carra, 2018). Under basal conditions only 5%–10% of SGs are degraded by granulophagy. However, autophagy-dependent SG degradation increases dramatically during stress and disease (Buchan et al., 2013; Mateju et al., 2017; Chitiprolu et al., 2018), which will be discussed later.

Autophagic clearance of SGs has been implicated by their recruitment of key autophagic proteins, such as p62 and LC3 (Buchan et al., 2013; Ganassi et al., 2016). A key study demonstrated that the molecular chaperone Cdc48 (human homologue: VCP), an ALS/FTD associated protein, is critical to autophagic degradation of SGs in yeast (Buchan et al., 2013). However, another study has shown that ULK1/2 localises to SGs where it phosphorylates VCP and increases its potential to drive SG disassembly in mammalian cells (Wang et al., 2019), which was not shared by other effectors of autophagy. Therefore, demonstrating both autophagy dependent and independent roles for classical autophagy proteins in SG clearance. Generally, selective autophagy involves the targeting of cargo by ubiquitination; this modification is recognized by the ubiquitin-associated domain of autophagy receptors (Conway et al., 2020), such as p62 and optineurin, which are also associated with ALS/FTD. However, one study has reported an alternative C9orf72 protein-mediated ubiquitin-independent granulophagy (Chitiprolu et al., 2018). Instead, symmetric arginine methylation of SG components by PRMT5 (protein arginine methyltransferase 5) was necessary for the selective targeting of SGs. This non-canonical signal was specifically recognized by p62 in a complex with C9orf72 via the Tudor protein SMN (survival motor neuron protein). Interestingly, impaired autophagy can also lead to anomalies in SG formation and morphology, which may be due to aberrant recruitment of defective ribosomal proteins (Seguin et al., 2014). These abnormal SGs contained noncanonical components, such as the 60S ribosomal subunit, which is normally absent. Furthermore, p62 and the autophagy receptor CALCOCO1 (also known as NDP52) have been implicated in autophagy-mediated degradation of retrotransposon RNA, localized to both RNA granules, P-bodies and SGs. This autophagy-mediated control in retrotransposon RNA degradation affects their insertion within the genome, which might be relevant for tempering somatic mosaicism (Guo et al., 2014). These selective mechanisms share the essential autophagy steps and machinery reviewed above, and so any impact on them may have a concomitant effect on RNA-selective autophagy degradation.

In addition, there are noncanonical roles for autophagy in RNA metabolism. **Secretory autophagy** is a newly defined autophagy function that bypasses the degradative process to allow the secretion of autophagic structures (Xu et al., 2018); of note, nucleic acids and RBPs have been found to be secreted by this pathway (Leidal et al., 2020). Interestingly, this process was also apparent in glial cells. Therefore, in addition to a known role for secretory autophagy in the release of aggregation-prone proteins (Lee J.-G. et al., 2016), the autophagy pathway may be implicated in non-cell autonomous control through excretion of nucleobases and RBPs (van Niel et al., 2018; Xu et al., 2018). LC3 has been reported to be responsible for the specific cargo loading of RBPs and small noncoding RNAs into vesicles to be secreted (Leidal et al., 2020). Interestingly, many RBPs are found to contain LC3-interacting (LIR) consensus motifs (Leidal et al., 2020). Furthermore, LC3 possesses a direct RNA-binding capacity through its own arginine-rich motif (Kraft et al., 2016), which has already been shown to play a role in the translational regulation of certain RNA (Zhou et al., 1997).

Another emerging role for autophagy in RNA metabolism is related to **RNA transport**, which is especially relevant for maintaining highly polarized cells such as neurons (Salogiannis and Reck-Peterson, 2017). The transport of RNA from neuronal soma to distal sites such as synapses allows local protein synthesis which is essential for the precise spatiotemporal control required for neuronal function. mRNAs associate with RBPs and are transported as RNA-protein complexes. Recent works have described a transport mechanism for RNA granules, known as “hitchhiking”, where they are indirectly transported along microtubules by docking onto endosomes, lysosomes or other membrane-bound organelles (Pohlmann et al., 2015; Gershoni-Emek et al., 2018; Cioni et al., 2019; Liao et al., 2019; Olgeiser et al., 2019). The ALS-related RBP Annexin A11 which is a component of SGs but also has phosphoinositide-binding capacity is reported to act as a molecular tether between RNA granules and lysosomes (Markmiller et al., 2018; Liao et al., 2019). Thus, due to the integration with the endocytic and lysosomal pathways, autophagy may also influence RNA transport.

## Dysfunctional Autophagy in Amyotrophic Lateral Sclerosis/Frontotemporal Dementia

**FIGURE 3:**
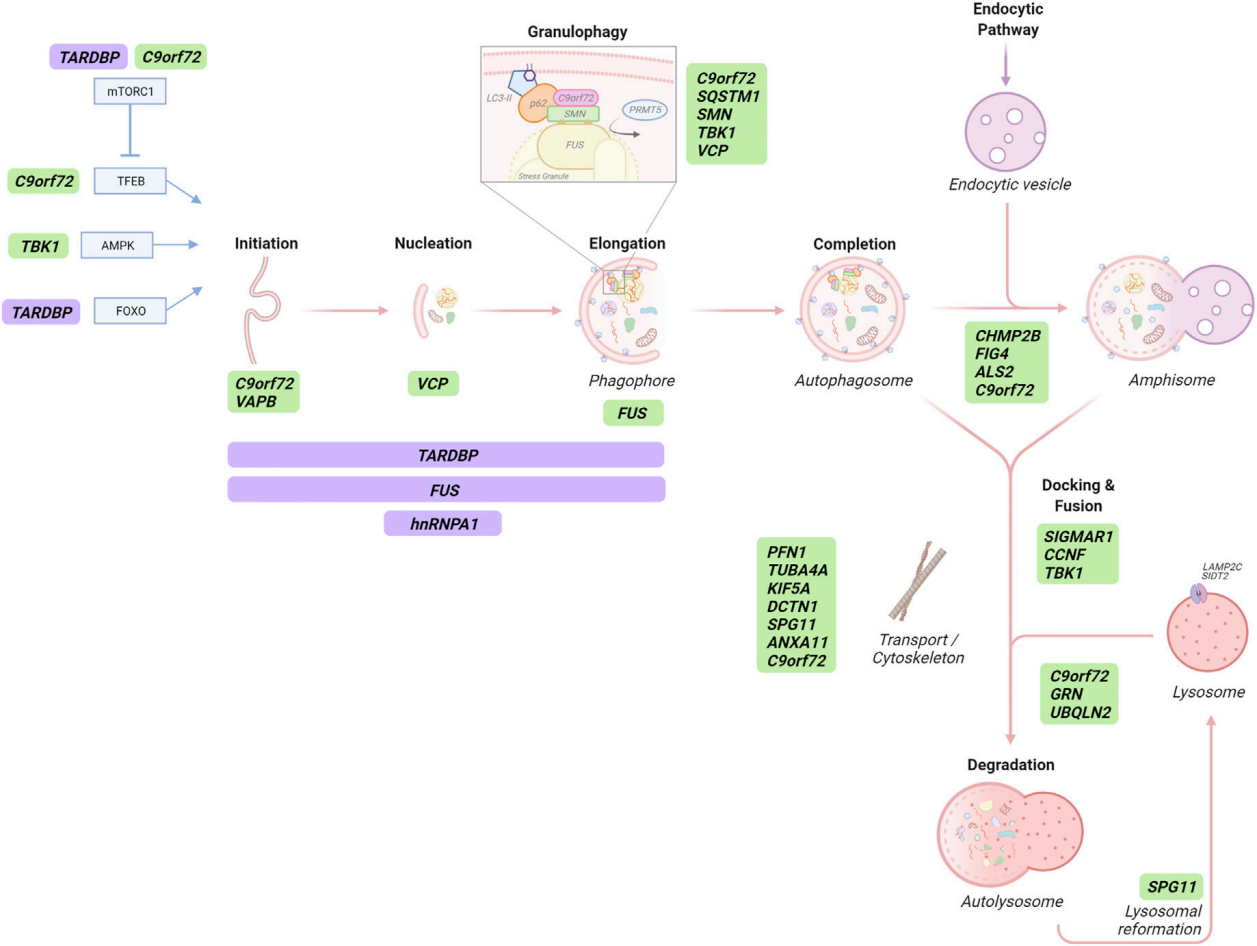
The role of ALS/FTD-associated genes in autophagy-dependent RNA catabolism. Several stages of the autophagy pathway, in addition to selective autophagic degradation of stress granules, are affected by ALS/FTD-linked gene (green) products, some of which are RBPs that transcriptionally and/or post-transcriptionally regulate autophagy-related proteins (purple). Autophagy initiation is transcriptionally regulated by TFEB, AMPK and FOXO which can be affected by C9orf72 protein and repeat RNA/DPRs, TBK1 and TDP43 (*TARDBP*), respectively. TFEB activity is modulated by mTORC1 which is also regulated by C9orf72 and TDP43 proteins. C9orf72, VAPB, VCP and FUS proteins play roles in different stages of autophagosome formation. Proteins participating in autophagosome formation are also post-transcriptionally regulated by TDP43, FUS and hnRNPA1 proteins. Selective SG degradation - termed granulophagy - can be mediated by C9orf72, p62 (*SQSTM1*), SMN, TBK1 and VCP proteins. CHMP2B, FIG4, ALS2 and the C9orf72 protein participate in the maturation of autophagosomes to form amphisomes in the intersection with the endocytic pathway. *SIGMAR1*, *CCNF* and *TBK1* gene products are required for appropriate autophagosome-lysosome membrane fusion. Retrograde transport of autophagic structures contributes to proper autophagy flux and it is affected by ALS/FTD-linked *PFN1*, *TUBA4A*, *KIF5A*, *DCTN1* and *SPG11* genes, coding for proteins of the vesicle transport machinery. Likewise, the C9orf72 protein is proposed to contribute vesicular trafficking, and annexin A11 (ANXA11) plays a role in RNA granule-lysosomal trafficking. Appropriate lysosomal degradative capacity of autophagic cargo is affected by ALS/FTD-linked proteins encoded by *C9orf72*, *GRN* and *UBQLN2*. Finally, spatacsin (*SPG11*) is involved in lysosomal reformation.

Several stages of the autophagy pathway, in addition to selective autophagy mechanisms, are affected by ALS/FTD-linked genes (Figure 3). Most of these are ubiquitous expressed, including in neurons and neuroglia, and although the majority of studies have focussed on the role of these genes in neurons, some neuroglia specific roles have been reported. Here, we summarize the roles of these ALS/FTD genes relative to the specific stages of autophagy.

### Autophagosome Formation

Since the discovery of the mutation in ALS/FTD, studies into loss of the **C9orf72** protein function (predominantly the long isoform) has been widely linked to regulation of the autophagy pathway. However, opposing effects have been reported. Several studies have shown that C9orf72 forms a stable complex with WDR41 (WD repeat-containing protein 41) and SMCR8 (Smith–Magenis chromosome regions 8) (Amick et al., 2016; Sellier et al., 2016; Sullivan et al., 2016; Ugolino et al., 2016; Yang et al., 2016; Zhang et al., 2018; Tang et al., 2020); this complex regulates the early steps of autophagy by interaction with the autophagy initiation complex, ULK1-FIP200-ATG13-ATG101 (Sellier et al., 2016; Sullivan et al., 2016; Webster et al., 2016; Yang et al., 2016; Ho et al., 2019). Perturbation of this interaction by reduced or knocked out C9orf72 has been associated with disrupted autophagy (Yang et al., 2016; Ho et al., 2019). However, other studies show that C9orf72 can negatively regulate autophagy (Ugolino et al., 2016; Yang et al., 2016; Liu et al., 2018; Ji et al., 2020; Wang M. et al., 2020) via upstream modulation of mTORC1 signalling (discussed below).

A mutation in the endoplasmic reticulum (ER)-mitochondria tethering protein **VAPB** (vesicle-associated membrane protein-associated protein B) is a rare cause of ALS (Nishimura et al., 2004) and causes a reduction in VAPB protein stability (Mitne-Neto et al., 2011). Interestingly, a decrease in VAPB protein is also observed in the spinal cord of people with sporadic ALS (Anagnostou et al., 2010). VAPB has been linked to several pathways involved in autophagy initiation. One function is exerted through binding to the mitochondrial protein PTPIP51 (protein tyrosine phosphatase-interacting protein-51) at ER-mitochondria contact sites, acting as a tether between the two organelles (de Vos et al., 2012; Stoica et al., 2014). ER-mitochondria contacts and the VAPB-PTPIP51 tethers have been shown to regulate autophagy by controlling ER-mitochondrial calcium transfer (Gomez-Suaga et al., 2017). Indeed, loss of either VAPB or PTPIP51 decreases ER-mitochondria contacts and increases autophagic flux, whereas overexpression of VAPB or PTPIP51 tightens the ER-mitochondria contacts and inhibits autophagosome formation (Gomez-Suaga et al., 2017). These effects are also seen in the context of selective autophagy during pathogen infection (Rimessi et al., 2020). VAPB has been proposed to exert a role in the recruitment of the autophagy initiation ULK1 complex during autophagosome formation through a direct interaction with ATG proteins (Zhao Y. P. et al., 2018). It also interacts with the ER-phagy receptor CALCOCO1, linking VAPB with early steps of this selective autophagy pathway (Nthiga et al., 2020). Mutant VAPB is also able to alter autophagic flux in multiple cell lines and a mutant knock-in mouse model (Larroquette et al., 2015), and accumulation of autophagy structures colocalising with VAPB has been reported in a *VAPB*-ALS patient fibroblasts and muscle biopsy (Tripathi et al., 2021). However, whether this increase in autophagy structures is related to the loss of function of wild-type VAPB or the mutant protein remains unknown.

### Maturation, Docking, and Fusion

**C9orf72** has been shown to interact with multiple Rab GTPases (Farg et al., 2014; Sellier et al., 2016; Webster et al., 2016; Yang et al., 2016; Aoki et al., 2017). Rab GTPases are major players in membrane trafficking events, including autophagy (Marat et al., 2011); like other GTPases, cycle between an inactive GDP-bound form in the cytoplasm and a membrane-associated GTP-bound active form. A guanine nucleotide exchange factor (GEF) facilitates the exchange of nucleotides and activates the Rab, which then interacts with downstream effectors to exert its biological function. Rab inactivation requires a GTPase-activating protein (GAP) to catalyse efficient nucleotide hydrolysis (Stenmark, 2009). The **C9orf72**-WDR41-SMCR8 complex was initially proposed to have GEF activity on Rab GTPases, such as Rab8A and Rab39B, resulting in their activation (Sellier et al., 2016; Yang et al., 2016; Iyer et al., 2018); both of which are implicated in autophagosome maturation (Pilli et al., 2012; Szatmári and Sass, 2014; Corbier and Sellier, 2017). Importantly, a constitutively active form of Rab39b, but not Rab8a, rescued autophagy defects caused by C9orf72 depletion in cultured primary mouse cortical neurons (Sellier et al., 2016). However, recent *in vitro* studies have suggested opposing GAP activity (Su et al., 2020; Tang et al., 2020; Nörpel et al., 2021); clearly an area of research that requires further clarification.

Autophagosome maturation is hampered by pathogenic variants in the ***ALS2*** gene as well. These variants have been identified in rare juvenile motor neuron diseases including ALS (Hadano et al., 2001; Yang et al., 2001). The *ALS2* gene encodes for Alsin 2, a GEF for the small GTPase Rab5, a key protein in the endocytic pathway (Otomo et al., 2003). Secondary knockout of *ALS2* displayed accumulation of autophagosome-like vesicles and accelerated disease progression in a mutant *SOD1*-ALS transgenic mouse model (Hadano et al., 2010).

Defects in the ESCRT system can cause various neurodegenerative diseases (Saksena and Emr, 2009). ESCRT-III proteins, including the ALS/FTD-associated protein **CHMP2B** (charged multivesicular body protein 2B) (Skibinski et al., 2005; Parkinson et al., 2006; Cox et al., 2010), are responsible for the sorting of cargoes and the completion of vesicular formation (Ugbode and West, 2021). *CHMP2B*-FTD is unusual in its lack of TDP-43 aggregates, presenting instead with inclusions positive for ubiquitin and p62 and lysosomal storage pathology (Holm et al., 2007; Clayton et al., 2015). The most common M178V mutation results in C-terminal truncation of CHMP2B; this results in loss of the MIT-interacting motif, which is required for interaction with Vps4, a critical ATPase in scission of vesicles into multivesicular bodies. CHMP2B mutant models and patient tissue display disrupted vesicle trafficking and accumulation of both endolysosomal and autophagy structures (Clayton et al., 2018; Ugbode and West, 2021).

A similar impact on the endo-lysosomal pathway can derive from ALS/FTD-associated loss-of-function variants in **FIG4** (Factor-induced gene 4) (Chow et al., 2009). FIG4 is a phosphoinositide phosphatase involved in (PI3P) generation (Ho et al., 2012); apart from its role in autophagy, PI3P has functions in several membrane tracking events such as membrane identity, endosomal maturation and degradation (Marat and Haucke, 2016). Loss or mutation of FIG4 results in lysosomal phenotypes in several disease models (Casterton et al., 2020) which according to recent work may be independent from its role as a phosphatase (Bharadwaj et al., 2016). *FIG4* null mice display p62-positive inclusions, of which most are found in astrocytes, highlighting the relevance of autophagic clearance in glial cells (Ferguson et al., 2009).

ALS/FTD can be caused by recessive mutations in the ***SIGMAR1*** gene, which encodes the sigma-1 receptor (sig-1R) (Luty et al., 2010). Although sig-1R participates in a broad array of biological functions (Aishwarya et al., 2021), reduced autophagy flux and accumulation of autophagic structures are characteristics of several cell and mouse models of *SIGMAR1*-ALS/FTD (Christ et al., 2020). Likewise, pharmacological activation or overexpression of sig-1R can increase autophagy flux *in vitro* and *in vivo* (Gregianin et al., 2016; Christ et al., 2019). Recently, loss of sig-1R or expression of ALS-associated mutants has revealed specific impairment of autophagosome-lysosome membrane fusion with concomitant autophagy flux defects (Yang et al., 2019).

Similarly, mutations in ***CCNF*** account for rare cases of familial ALS-FTD (Williams et al., 2016). Cyclin F, encoded by *CCNF* gene, is one of the components of an E3 ubiquitin-protein ligase complex and ALS/FTD-associated mutations have been found to impair autophagosome-lysosome fusion through direct binding and increased ubiquitylation of p62/SQSTM1 (Lee et al., 2018).

Finally, in neurons, autophagosomes in distal processes are required to be transported toward the soma where mature acidic lysosomes are predominantly located, and thus impairments in retrograde transport can contribute to defective autophagy flux (Nixon, 2013). Axonal transport deficiency is observed early in many different ALS/FTD models, and therefore may play an important role in the initiation of disease (de Vos and Hafezparast, 2017). Indeed, several pathogenic variants in genes encoding vesicular transport machinery proteins, such as ***PFN1*** *(*profilin*)* (Chen et al., 2013; Ingre et al., 2013), ***TUBA4A*** (tubulin isotype α4a) (Smith et al., 2014), **KIF5A** (kinesin family member 5A) (Nicolas et al., 2018) and ***DCTN1*** *(*dynactin subunit 1) (Münch et al., 2004), in addition to the cytoskeleton component ***SPG11*** (spatacsin) (Orlacchio et al., 2010), are directly associated to ALS. Defective transport linked to these genes might damage autophagy by impairing anterograde movement of lysosomes, lysosomal biogenesis and/or autophagosome clearance; indeed, mutations in dynactin have been linked to an impairment in autophagosome-lysosome fusion due to redistribution to the cell periphery (Farías et al., 2017; Laird et al., 2008; Münch et al., 2004; Yu et al., 2018). The **C9orf72** protein may also play a role in both vesicular trafficking and cytoskeleton organization: by interaction with cofilin 1 and modulation of the small GTPases ARF6 and RAC1, C9orf72 has been associated to actin dynamics and axon outgrowth in cultured motor neurons (Sivadasan et al., 2016).

### Lysosomal Degradation

A role for the **C9orf72** protein in the lysosomal-autophagy pathway has been identified by its lysosomal localization during amino acid starvation; this localization is driven by an interaction of WDR41 within the C9orf72-WDR41-SMCR8 complex with the lysosomal amino acid transporter PQLC2 (Amick et al., 2016; Amick et al., 2018; Amick et al., 2020). In agreement, C9orf72 knockdown in cells can affect both autophagy induction and flux (Yang et al., 2016). Studies support a role for C9orf72 at the lysosome in degradation and exocytosis, due to impaired autolysosome acidification, and also transcriptional regulation (see below) and potentially lysosomal reformation (Amick et al., 2016; Corrionero and Horvitz, 2018; Zhang et al., 2018; Shao et al., 2020). Of note, although early studies reported co-localization of C9orf72 protein with several organelles of the endocytic pathway, the nucleus, the Golgi apparatus, neurites, growth cones and RNA granules (Renton et al., 2011; Farg et al., 2014; Atkinson et al., 2015; Aoki et al., 2017; Chitiprolu et al., 2018; Shi et al., 2018), a validation study of the commercial antibodies used in these studies showed that many cannot specifically recognize C9orf72 protein (Laflamme et al., 2019); however, the antibodies determined to be specific still localised C9orf72 to lysosomal and autophagy structures. This study also confirmed high expression of C9orf72 in immune cells, which links in with the strong phenotype of autoimmunity and inflammatory disease in C9orf72 knockout mice (Balendra and Isaacs, 2018).

Indeed, several studies have demonstrated that C9orf72 protein is required for proper macrophage/microglial function, with abnormal lysosomal accumulation in knockout cells (O’Rourke et al., 2016; Zhang et al., 2018; Shao et al., 2020; Lall et al., 2021). This is consistent with the microglial hyperactivation and astrogliosis that have been described in *C9orf72* ALS/FTD *post-mortem* brain and cerebrospinal fluid (Haidet-Phillips et al., 2011; Brettschneider et al., 2012; Cooper-Knock et al., 2012; Oeckl et al., 2019). A large body of evidence now supports the notion that glial cells can contribute to ALS/FTD (Meyer et al., 2014; Madill et al., 2017). As mentioned above, microglial autophagy is crucial for synaptic homeostasis in mice (Kim et al., 2017). Indeed, the microglia in both pan and microglia-specific C9orf72-knockout mice become activated, exhibit enhanced synaptic pruning and accumulate lysosomal material (Lall et al., 2021). On the other hand, the autophagy machinery has been related to the non-canonical autophagy function of secretory autophagy, of special relevance in astrocytes, the glial cells which provide trophic support for neurons (Verkhratsky et al., 2016). Indeed, dysfunctional autophagy in astrocytes is known to contribute to neurodegeneration in a lysosomal store disorder by impairing their ability to metabolically support neurons (di Malta et al., 2012; Sung and Jimenez-Sanchez, 2020). Regarding *C9orf72*-ALS/FTD, a recent study has associated the mutation to altered astrocytic extracellular vesicle secretion of micro RNAs (miRNAs) that are involved in the regulation of axonal maintenance genes and restoration of miRNA activity could partially ameliorate toxicity of conditioned media from *C9orf72* patient derived astrocytes or motor neurons (Varcianna et al., 2019). Since miRNAs can also act as regulators of autophagy (Shah et al., 2018), it would be interesting to analyse a possible non-autonomous control of neuronal autophagy by *C9orf72* mutant astrocytes. Additionally, the mechanisms underlying *C9orf72* mutant astrocyte-mediated neurotoxicity may arise from secretion of neurotoxic factors (Haidet-Phillips et al., 2011), such as inflammatory cytokines also via the secretory autophagy pathway (Colombo and Farina, 2016). Accumulation of p62 and increased secretion of lysosomal components are also observed in *C9orf72* deficient macrophages, which promote a pro-inflammatory state and altered immune responses (Zhang et al., 2018; Shao et al., 2020), suggesting a role for the encoded C9orf72 protein. Collectively, these studies evidence the contribution of defective glial autophagy and non-cell autonomous patho-mechanisms via impairments in lysosomal function from loss of C9orf72 protein in *C9orf72*-ALS/FTD.

The **C9orf72** protein has also been linked to inappropriate sorting of lysosomal hydrolase precursors by an effect on retromer trafficking via interaction with Rab7L1 (Burd and Cullen, 2014; Aoki et al., 2017). The retromer is involved in the retrograde transport of transmembrane proteins from the late endosomes to the trans-Golgi network (TGN). This includes the mannose 6-phosphate receptor (M6PR) which cycles between the TGN and endosomes to deliver lysosomal hydrolases through the endocytic pathway into lysosomes. Downregulation of *C9orf72* caused dysfunctional retromer transport of M6PR and impaired autophagosome accumulation in *C9orf72*-ALS/FTD patient-derived fibroblasts (Aoki et al., 2017). In agreement, a recent study has shown a correlation between deficits in retromer and lysosomal functions and reduced synaptic vesicular trafficking in *C9orf72* patient and *C9orf72* knockout iPSC-derived motor neurons (Shi et al., 2018).

Haploinsufficiency of progranulin (PGRN) due to loss-of-function mutations in the ***GRN*** gene is a leading genetic cause of pure FTD (Baker et al., 2006; Cruts et al., 2006; Schymick et al., 2007; Cannon et al., 2013). Multiple functions have been described for progranulin, but evidence supports a major role for this glycoprotein in regulating different aspects of lysosomal biology (Kao et al., 2017). Progranulin can both facilitate lysosomal acidification and regulate lysosomal protein levels and gene expression (Tanaka et al., 2017). Reduction of progranulin resulted in increased lysosomal gene expression and protein levels, the latter of which included mature Cathepsin D, in agreement with its accumulation in PGRN-FTD patient brain (Tanaka et al., 2017). Knockdown of progranulin has however been shown to both decrease and increase autophagic flux (Liu et al., 2015; Chang et al., 2017; Elia et al., 2019). A recent work has proposed an additional loop in which the level of progranulin itself is regulated by autophagy, as a substrate (Elia et al., 2019). Thus, there is a complex interplay of progranulin with the autophagy-lysosomal pathway. Interestingly, both overexpression and knockdown of progranulin has been reported to result in the accumulation of insoluble TDP-43 and varying between cell lines, suggesting that selective autophagy may also be compromised in PGRN-FTD (Tanaka et al., 2017; Elia et al., 2019). As other ALS/FTD-associated proteins, progranulin is broadly expressed by many cell types, including neurons, astrocytes and prominent in activated microglia, regulating neuron and immune functions (Petkau and Leavitt, 2014; Elia et al., 2019). Indeed, a still not well-understood neuro-immunomodulatory action has been postulated for progranulin in microglial activation (Chitramuthu et al., 2017). Progranulin deficiency leads to alterations in lysosomal function that drive increased production of complement proteins and enhanced synaptic pruning by microglia (Lui et al., 2016). These findings suggest that progranulin-mediated neurodegeneration may be partially caused by lysosomal dysfunction and subsequent aberrant microglial activation.

Missense mutations in ***UBQLN2*** gene cause X-linked ALS/FTD (Deng et al., 2011), and cytoplasmic inclusions of the encoded protein ubiquilin-2 have been described in degenerating motor neurons broadly in familial and sporadic ALS (Fecto and Siddique, 2011). Disruption of both proteasomal degradation and autophagy have been proposed as pathogenic mechanisms for mutant ubiquilin-2 (Casterton et al., 2020). Although ubiquilin-2 can indirectly interact with LC3 (Rothenberg et al., 2010), it seems to predominantly act instead in later stages of autolysosome degradation; two recent works have shown that ubiquilin 2 is required for lysosomal acidification, exerting a regulatory role on V-ATPase function via interaction with different subunits of the V-ATPase pump (Şentürk et al., 2019; Wu et al., 2020).

Finally, loss-of-function mutations in the ***SPG11*** gene, encoding for the cytoskeleton component spatacsin, are a cause of juvenile-onset ALS (Orlacchio et al., 2010) and have been found to play a role during lysosomal reformation (Chang et al., 2014).

### Transcriptional and Post-Transcriptional Regulation

Although TDP-43 and FUS-mediated pathogenicity is established through direct deregulation of RNA homeostasis (Ling et al., 2013), they also have roles as autophagy regulators. Several studies have implicated **TDP-43** in autophagy regulation post-transcriptionally. TDP-43 can bind to and regulate the mRNAs of multiple autophagy-related genes including *ATG7*, *SQSTM1/p62*, *VCP*, *ATG4B*, *DCTN1* and the mTORC1 key component Raptor/RPTOR (Bose et al., 2011; Ling et al., 2015). However, the effects of this mRNA regulation on autophagy is complex; for example, although downregulation of Raptor by TDP-43 depletion causes mTORC1 inhibition and TFEB activation which signals for autophagy induction, reduced TDP-43 also causes an accumulation of autophagosomes due to impaired autophagosome-lysosome fusion, which was suggested to be due to downregulation of dynactin 1 (*DCTN1*) (Xia et al., 2016). TDP-43 dysfunction results in the dysregulated expression of other ALS-associated proteins which also have roles in autophagy, including FUS, progranulin (GRN) and ataxin-2 (ATXN2). Additionally, TDP-43 can mediate the activation of FOXO transcription factors during proteostatic stress and induce the expression of genes involved in protein quality control (Zhang T. et al., 2014); FOXO regulates genes relating to both proteasomes and autophagy (Mammucari et al., 2007; Zhao et al., 2008; van der Vos et al., 2012).

Regarding the role of **FUS** in autophagy regulation, a recent study has found that the mRNA and protein levels of key genes involved in initial steps of the autophagy pathway, including *FIP200*, *ATG16L1* and *ATG12*, are significantly lowered in *FUS* depleted cells (Arenas et al., 2021). Moreover, previous studies have described defective autophagy in different ALS-linked *FUS*-disease models, suggesting additional roles for FUS in the autophagy process. Expression of mutant FUS disrupted autophagy induction in neuronal cells by interfering with early autophagosome formation in a Rab1-dependent manner (Soo et al., 2015). Intriguingly, the expression of wild-type FUS also impaired autophagosome formation and maturation in neuronal like cells (Ling et al., 2019). Thus, further studies investigating the molecular mechanisms involved in FUS-mediated inhibition of autophagy will be needed.

Other ALS/FTD-related RBPs governing the autophagy process at a post-transcriptional level include **hnRNPA1**, which binds to the *Beclin1* mRNA and positively regulates its expression (Ji et al., 2019). However, whether these findings suggest a mechanism by which pathogenic variants in *HNRNPA1* might affect autophagy initiation, requires further investigation. Conversely **VCP** regulates autophagy initiation by Beclin-1 protein directly by promoting its deubiquitination and regulating the assembly of the phosphatidylinositol-3-kinase (PI3K) lipid signalling complex (Hill et al., 2021).

Loss of function studies have implied that the **C9orf72** protein can also act as an indirect negative regulator of autophagy; knockdown or knockout of C9orf72 has been reported to cause mTORC1 inactivation, resulting in the nuclear translocation of the transcription factor TFEB and autophagy induction in several studies (Ugolino et al., 2016; Yang et al., 2016; Liu et al., 2018; Ji et al., 2020; Wang M. et al., 2020). Indeed, two new studies have provided additional evidence for this negative regulation of mTORC1-dependent autophagy regulation through the interaction of C9orf72 protein with Rag GTPases on the lysosomal membrane, which recruit TFEB to the lysosome for phosphorylation by mTORC1 (Ji et al., 2020; Wang M. et al., 2020). Adding more complexity to the picture, a dual role in autophagy regulation has also been suggested by a study that showed that loss of C9orf72 impairs mTORC1-mediated autophagy induction while inducing autophagy flux under basal conditions (Yang et al., 2016). Likewise, a recent study showed that C9orf72 protein overexpression may enhance autophagy initiation basally but impair autophagy under conditions of stress (Cali et al., 2019).

Furthermore, ***C9orf72*** repeat RNA binds and sequesters several RBPs that have roles in gene regulation (reviewed by McEachin et al., 2020). Of note to autophagy, a recent study linked the known *C9orf72* repeat RNA and DPR-mediated disruption of nucleocytoplasmic transport (Kim and Taylor, 2017; Solomon et al., 2021) to impaired autophagy regulation by TFEB; expression of *C9orf72* repeats reduced TFEB translocation to the nucleus resulting in defective autophagy and accumulation of protein aggregates in *Drosophila*, an effect that appeared to be predominantly driven by repeat RNA, with only a small effect from the DPR poly(GA) (Cunningham et al., 2020)*.* Consistently, this study described nuclear TFEB depletion in the motor cortex of ALS patients, corroborating a previous study (Wang et al., 2016). Together it is clear that the *C9orf72* mutation can cause multiple effects on autophagy, predominantly via haploinsufficiency of the protein but also by gain of function repeat RNA and DPR mechanisms, and further investigations will be required to fully determine their roles in FTD/ALS pathogenesis.

## Dysfunctional Autophagy-Dependent RNA Homeostasis in Amyotrophic Lateral Sclerosis/Frontotemporal Dementia

Regarding autophagy-mediated RNA homeostasis in ALS/FTD, the majority of the evidence comes from studies on SGs. As mentioned previously, the contribution of autophagy to the clearance of SGs under basal conditions is low but increases significantly upon cellular stress or in disease (Ganassi et al., 2016; Mateju et al., 2017; Mediani et al., 2021). Indeed, accumulating evidence suggests that SGs are targeted for selective autophagic degradation when granules become pathological (Sun et al., 2020). Although, SGs can be part of a pro-survival mechanism, chronic SGs prevent the normal functionality of a cell. If not efficiently cleared, persistent SGs may form the seed for aggregation of RBPs (Baradaran-Heravi et al., 2020), and chronic optogenetic induction of SGs has been shown to drive neurotoxicity (Zhang et al., 2019). Thus, under disease conditions the selective autophagic degradation of SGs may play a crucial role.

Mutations are found in several ALS/FTD genes encoding proteins involved in selective autophagy. The ALS-associated autophagy receptor **p62** is recruited to SGs upon cellular stress (Chitiprolu et al., 2018; Turakhiya et al., 2018); **TBK1** (TANK-binding kinase 1; (Cirulli et al., 2015; Freischmidt et al., 2015)) phosphorylates p62 (Pilli et al., 2012; Matsumoto et al., 2015) and another autophagy receptor - optineurin (Matsumoto et al., 2015), which enhances affinity to cargo and promotes selective autophagy of damaged mitochondria or intracellular pathogens (Richter et al., 2016). There have also been reports that **TBK1** regulates upstream components of the autophagy signalling cascade such as AMPK (Zhao Y. G. et al., 2018) or the autophagosome-localized SNARE protein Syntaxin-17 (Kumar et al., 2019). Interestingly, SMCR8, a **C9orf72** protein binding partner, is also a substrate of TBK1 (Sellier et al., 2016), which may therefore have implications in pathogenesis from the range of roles of the C9orf72-WDR41-SMCR8 complex in autophagy. As explained above, C9orf72, p62 and **SMN** are also involved in an alternative ubiquitin-independent granulophagy dependent on symmetric arginine methylation of SG components. Indeed, *C9orf72*-ALS patient cerebellar tissue exhibits an accumulation of symmetrically arginine-methylated proteins (Chitiprolu et al., 2018), suggesting deficient turnover of SGs, and deregulation of autophagy is observed in cell culture and animal models of SMN-associated neurodegeneration (Garcera et al., 2013; Custer and Androphy, 2014). Although in most cases, studies have not investigated a direct link between granulophagy and ALS/FTD associated autophagy genes, disease associated mutations in **VCP** (Gitcho et al., 2009; Johnson et al., 2010) have been shown to induce persistent SGs which also contain TDP-43 (Buchan et al., 2013; Gwon et al., 2021) and depletion or inhibition of VCP impairs SG formation (Seguin et al., 2014). Interestingly, the *C9orf72* DPR poly(GA) has been shown to sequester VCP (Božič et al., 2021), which may similarly inhibit VCP’s function in selective autophagy and/or SG dynamics. **VAPB** patient muscle biopsies also contain VAPB aggregates that colocalise with TDP-43 and the SG protein TIAR1 (Tripathi et al., 2021), suggesting impaired autophagy-dependent SG clearance.

Perturbed RNA granule dynamics has drawn much attention from the ALS-FTD field in recent years; pathological granules transition from a rapidly reversible liquid state to a more solid gel-like state which renders them less dynamic and persistent (Wolozin and Ivanov, 2019; Solomon et al., 2021). Investigation of the ***C9orf72*** arginine-rich DPR interactomes found a wide number of RBPs and were enriched in those involved in membraneless organelles including SGs components and major ALS/FTD RBP proteins, such as TDP-43 and hnRNPA1 (Lee K.-H et al., 2016; Boeynaems et al., 2017; Chew et al., 2019). Consequently, these arginine-rich DPRs—both poly(GR) and poly(PR) - have been shown to alter SG composition and dynamics by disrupting multivalent interactions and promoting the formation of pathological SGs (reviewed by Solomon et al., 2021). Similarly, mutants of several ALS/FTD-related RBPs, such as **FUS** and **TDP-43**, promote the formation of poorly dynamic SGs (Dewey et al., 2011; Baron et al., 2013; Murakami et al., 2015; Patel et al., 2015; Gopal et al., 2017; Ding et al., 2021). SGs can also accumulate defective ribosomal proteins (Seguin et al., 2014) and misfolded proteins, such as mutant **SOD1** (Mateju et al., 2017), which can drive them into a pathological state (Alberti and Carra, 2018). In line with their role in the clearance of pathological SGs, persistent mutant FUS-positive SGs were found to co-localize with LC3-II (Ryu et al., 2014). Pharmacological activation of autophagy can also reduce FUS-and TDP-43-related cytotoxicity, which was associated with a reduction in the number of TDP-43- or FUS-positive SGs (Wang et al., 2012; Boyd et al., 2014; Ryu et al., 2014; Marrone et al., 2019). The ALS/FTD protein **ubiquilin 2** can also be recruited to SGs where its interaction with the RBP FUS is proposed to cause FUS dissociation from SGs, a process which is impaired in disease mutants (Alexander et al., 2018; Dao et al., 2018). **Optineurin** can also modulate SG dynamics and clearance in autophagy dependent and independent ways; in the latter, reduced optineurin or disease mutants lead to an upregulation of TIA1 expression causing reduced SG clearance and accumulation of ubiquitinated TDP-43 (Kakihana et al., 2021). Furthermore, SGs also participate in autophagy regulation by recruiting signalling molecules, such as the components of the mTORC1 complex, raptor and mTOR; this association regulates TORC1 inactivation-reactivation during cell stress and recovery (Takahara and Maeda, 2012; Yan et al., 2012; Wippich et al., 2013). Sequestration of these signalling molecules or other RBPs into poorly dynamic SGs may compromise the autophagy–lysosomal pathway indirectly.

Finally, autophagic degradation of SGs and other RNA granules also relies on proper trafficking. As noted previously, ALS-associated variants in ***ANXA11*** have been shown to disrupt RNA granule-lysosome docking, impeding their transport in neurons (Liao et al., 2019) and ANXA11 mutants can impede calcium homeostasis and stress granule disassembly (Nahm et al., 2020).

In summary, although thus far relatively few studies have connected autophagy and RNA homeostasis directly, there is clear evidence for a significant impact of autophagy on RNA homeostasis in FTD/ALS, particularly in relation to the clearance of pathological SGs.

## Discussion

Current knowledge reviewed here has established a multilayer understanding of autophagy regulation as well as novel non-canonical functions for the autophagy machinery. An emerging and less-well understood area of autophagy research involves its role in RNA metabolism. Here we discussed new evidence of specialized mechanisms for selective autophagy-mediated RNA degradation and other autophagy functions also affecting RNA homeostasis. We have also described how RBPs might not serve only as autophagic cargos but also exert regulatory functions on the autophagy process, adding additional complexity to the RNA-autophagy interplay.

This complex autophagy-RNA interplay is well evidenced in ALS/FTD, where dysregulated RNA homeostasis and autophagy defects are major interconnected disease mechanisms. We discussed here the role of ALS/FTD-associated proteins in the different stages of autophagy, disrupting general autophagy but also specific RNA-mediated autophagy, with SGs as major substrates in this process. The evidence presented here reinforce a unifying connection between many ALS/FTD genes and suggest a model in which pathogenic variants that dysregulate autophagic clearance and promote an accumulation of pathological RNA granules and drive cytoplasmic protein aggregation culminate in ALS/FTD.

The mechanisms associated to the *C9orf72* mutation have been a focus of the ALS/FTD research field as major known cause of disease but also for its intriguing complex disease mechanisms. *C9orf72* mutation studies nicely illustrate the relevance and complexity of the autophagy–RNA homeostasis interplay in health and disease. As reviewed above, as part of the C9orf72-WDR41-SMCR8 complex, the C9orf72 protein is involved in the initiation of autophagy and localised to the lysosome. Here, loss of C9orf72 results in inappropriate sorting of hydrolase precursors, impaired synaptic vesicle recycling, lysosomal accumulation, and alterations in glial secretomes. This latter finding has wide implications on the neuroimmune system and may contribute to neuroinflammation and non-cell autonomous neurotoxicity. It can also impact gene regulation of autophagy via its inactivation of mTORC and autophagosome maturation and trafficking via interaction with cofilin. Through these roles haploinsufficiency of the C9orf72 protein can affect RNA homeostasis by selective autophagy. However, the C9orf72 protein additionally impacts granulophagy by a direct role in a ubiquitin-independent pathway targeting arginine-methylated SG substrates for degradation.

In *C9orf72*-FTD/ALS, gain-of-function mechanisms emanating from the repeat are proposed to be the dominant cause of neurodegeneration (Mizielinska et al., 2014; Moens et al., 2019), and both repeat RNA and DPRs can affect the autophagy-RNA homeostasis interplay. Dysfunctional nucleocytoplasmic transport from both the repeat RNA and DPRs may impact the nuclear translocation of transcription factors that regulate autophagy genes (Kim and Taylor, 2017; Solomon et al., 2021); indeed, the master regulator TFEB is affected by expression of the *C9orf72* repeat in Drosophila and cell models, an effect that appeared to be predominantly driven by repeat RNA with only a small effect from poly(GA) (Cunningham et al., 2020). The arginine rich DPRs bind to and disrupt a wide range of membraneless organelles (Boeynaems et al., 2017; Lee K.-H et al., 2016), including the nuclear pore through which all nucleocytoplasmic transport occurs, thus may contribute to the aforementioned disruption. However, as described above the arginine rich DPRs also bind SG components and perturb SG dynamics, which may result in the persistent pathological SGs that are targeted for degradation by autophagy. As noted, these effects are associated with the arginine rich DPRs which may mimic other arginine rich motifs such as the RGG motif which is common to RBPs involved in RNA homeostasis; similarly to the arginine rich DPRs, this motif permits interaction with both proteins and nucleic acids (Rajyaguru and Parker, 2012). This motif also undergoes regulation by arginine methylation—a signal already described to target cargo to SGs for autophagic degradation involving the C9orf72 protein (Chitiprolu et al., 2018). Indeed, the arginine rich DPRs also undergo arginine methylation, with different methylation types regulating its pathogenicity (Sakae et al., 2018; Gittings et al., 2020). Finally, the aggregates formed by the *C9orf72* repeat RNA and DPRs can sequester proteins, for example the sequestration of VCP by poly(GA) (Božič et al., 2021), which promotes further dysfunction by these pathways. Gain-of-function DPR pathomechanisms can also be exacerbated by the coincident loss of C9orf72 protein levels in *C9orf72*-ALS/FTD which reduce their autophagic clearance. Indeed, complete knockout or reduction of C9orf72 exacerbates reduced survival in *C9orf72* repeat expansion models in neuron-like cell lines, zebrafish and mice (Shao et al., 2019; Boivin et al., 2020; Zhu et al., 2020) and from DPR expression in control patient induced motor neurons (Shi et al., 2018). Conversely C9orf72 protein expression can ameliorate reduced lysosomal number, DPR accumulation and degeneration in *C9orf72*-ALS/FTD patient induced motor neurons (Shi et al., 2018).

Although studies have identified an effect of loss of C9orf72 protein function on clearance of DPRs, it has not yet been investigated whether it could also affect the degradation of *C9orf72* repeat RNA, given the emerging evidence for the role of autophagy in RNA catabolism. Indeed, the mechanisms for the degradation of repeat RNA in repeat expansion disorders are still poorly understood. A recent study has revealed a role for the RNA exosome, which is generally involved in RNA quality control, in the degradation of the *C9orf72* sense and antisense repeat transcripts (Kawabe et al., 2020). The RNA exosome exerts its functions in an autophagy/lysosomal-independent manner. Instead, the catalytic component EXOSC10 promotes the substrate loading into the tunnel of the barrel-like core of the RNA exosome complex for its degradation (Kilchert et al., 2016). However, additional mechanisms for *C9orf72* expanded G_4_C_2_ repeat RNA are tempting to hypothesis when the RNautophagy receptors LAMP2C and SIDT2 have already shown to have binding preference for stretches of consecutive guanosines (Hase et al., 2015). Indeed, SIDT2 was found to interact with expanded CAG repeats in exon1 of the *HTT* transcript, which code for polyglutamine-expanded proteins, linked to Huntington’s disease. Importantly, overexpression of SIDT2 promoted degradation of *HTT* expanded transcripts, reducing the levels of polyglutamine-expanded huntingtin aggregates. Thus, *C9orf72*-derived expanded G_4_C_2_ repeat RNA could be directed to lysosomes for RNautophagy degradation in a similar manner. Although, in the case of the *C9orf72* repeat RNA, hairpin and G-quadruplex structures will need to be considered (Fratta et al., 2012; Haeusler et al., 2014). It is also of note that the C9orf72 protein itself is a substrate for autophagy in a cell-type dependent manner (Leskelä et al., 2019).

In summary, both synergistic and feedback loops in autophagy and RNA homeostasis contribute to *C9orf72*-ALS/FTD pathogenesis, from gain and loss of function mechanisms. Loss of the C9orf72 protein may impact several stages of autophagy as well as granulophagy directly, which can exacerbate gain of function mechanisms by compromising DPR protein and potentially repeat RNA clearance. Then, the DPRs and repeat RNA can also impact autophagy and RNA homeostasis themselves, through effects on transcription factors, SG dynamics and potentially RNautophagy. Interestingly, a recent genome-wide data study has revealed that autophagy genes are significantly associated with ALS risk in *C9orf72* expansion carriers, supporting a modifying role for autophagy from a genetic perspective as well (Saez-Atienzar et al., 2021). But it does not end there, as with the majority of ALS and a large proportion of FTD cases, the *C9orf72* mutation is associated with pathology of the RBP TDP-43, and RBPs can also modulate autophagy. Indeed, the ALS/FTD-associated RBPs TDP-43, FUS and hnRNPA1 can all regulate the transcription of autophagy-related genes, with TDP-43 also able to modulate the transcription factor FOXO and FUS to both initiation and maturation of autophagosomes. Conversely, many ALS/FTD-linked genes can impact autophagy and RNA homeostasis via contribution to granulophagy, with evidence for the proteins encoded by *SQSTM1* (p62) and *VCP* playing crucial roles.

Another point to consider is that multiple different cell types contribute to ALS/FTD pathologies (Ragagnin et al., 2019; Vahsen et al., 2021) and the autophagic-RNA homeostasis interplay may vary between these. Indeed, accumulation of insoluble protein, often RBP, inclusions are found in both neurons and neuroglia of ALS/FTD post-mortem tissue indicative of impaired protein clearance (Neumann et al., 2007). Of particular interest is also the role for secretory autophagy in the release of extracellular vesicles (di Malta et al., 2012; Verkhratsky et al., 2016) and excretion of nucleobases and RBPs (Shah et al., 2018; Varcianna et al., 2019; Leidal et al., 2020) from glial cells and their potential contribution to non-cell autonomous patho-mechanisms in ALS/FTD. However, as much of the research into the interplay of autophagy and RNA catabolism is very recent, further studies will be required to dissect contributions of different cell types to pathology via these pathways, which has implications for therapeutic targeting.

Indeed, an important point to consider is that these studies have opened a new avenue of research searching for therapeutic approaches for these fatal diseases. Autophagy has long been explored as a therapeutic target for ALS/FTD with contradictory results, depending on the ALS/FTD causing gene or disease-model. For instance, pharmacologically autophagy activation exacerbated neurotoxicity in *Drosophila TARDBP* (TBPH) knockout and SOD1^G93A^ mice (Zhang et al., 2011; Hsueh et al., 2016; Xia et al., 2016; Zhou et al., 2017). While a neuroprotective role for autophagy induction was reported in several *FUS* and *TARDBP* disease models (Caccamo et al., 2009; Wang et al., 2012; Burkhardt et al., 2013; Barmada et al., 2014; Boyd et al., 2014; Cheng et al., 2015) or even in the same SOD-1 mouse model (SOD1^G93A^) (Fornai et al., 2008; Mancuso et al., 2014; Zhang T. et al., 2014). However thus far relatively little consideration has been given to the impact of targeting RBPs on autophagy, and we are not aware of any translational approaches for autophagy-dependent RNA catabolism in the ALS/FTD field as of yet.

In summary, we have reviewed the wide evidence for the contribution of ALS/FTD-related proteins and RNA to disrupt the different stages of autophagy. Through this dysfunction they have the potential to impact RNA catabolism in addition to their roles in direct RNA homeostasis deregulation. Of particular note is the interplay at the level of SG clearance (granulophagy), as alterations in SG dynamics and the contribution to disease pathology is of major interest to the field. Thus, future research may focus on more specific pharmacological interventions targeting pathological SGs for clearance via autophagy-dependent mechanisms. In addition, increasing our understanding of the molecular mechanisms of autophagy-dependent RNA homeostasis and the context in which these processes become dysfunctional in disease will be beneficial to design effective therapeutics particularly in ALS/FTD.
